# Socioeconomic inequality in violent behaviors, life dissatisfaction, and self-rated health in pediatric population: the CASPIAN-V study

**DOI:** 10.1186/s12888-022-04122-8

**Published:** 2022-08-02

**Authors:** Mostafa Qorbani, Mostafa Amini Rarani, Amir Kasaeian, Shirin Djalalinia, Kourosh Nouri, Hadith Rastad, Ehsan Shahrestanaki, Mohammad Esmaeil Motlagh, Ramin Heshmat, Roya Kelishadi

**Affiliations:** 1 Non-communicable Diseases Research Center, Alborz University of Medical Sciences, Karaj, Iran; 2 Endocrinology and Metabolism Research Center, Endocrinology and Metabolism Clinical Sciences Institute, Tehran University of Medical Sciences, Tehran, Iran; 3 Health Management and Economics Research Center, Isfahan University of Medical Sciences, Isfahan, Iran; 4 Hematology, Oncology and Stem Cell Transplantation Research Center, Research Institute for Oncology, Hematology and Cell Therapy, Tehran University of Medical Sciences, Tehran, Iran; 5 Digestive Diseases Research Center, Digestive Diseases Research Institute, Tehran University of Medical Sciences, Tehran, Iran; 6 Inflammation Research Center, Tehran University of Medical Sciences, Tehran, Iran; 7 Development of Research & Technology Center, Ministry of Health and Medical Education, Tehran, Iran; 8 Student Research Committee, Alborz University of Medical Sciences, Karaj, Iran; 9 Social Determinants of Health Research Center, Alborz University of Medical Sciences, Karaj, Iran; 10 Department of Epidemiology, School of Public Health, Iran University of Medical Sciences, Tehran, Iran; 11 Department of Pediatrics, Ahvaz Jundishapur University of Medical Sciences, Ahvaz, Iran; 12 Chronic Diseases Research Center, Endocrinology and Metabolism Population Sciences Institute, Tehran University of Medical Sciences, Tehran, Iran; 13 Department of Pediatrics, Child Growth, and Development Research Center, Research Institute for Primordial Prevention of Non-communicable Disease, Isfahan University of Medical Sciences, Isfahan, Iran

**Keywords:** Adolescents, Bully, Victim, Children, Life dissatisfaction, Self-rated health

## Abstract

**Background:**

Bullying, being a victim of violent behaviors, life satisfaction (LS) and self-rated health (SRH) in children and adolescents, all have consistently been recognized as vital factors in school performance and future individual life.

**Methods:**

This cross-sectional data secondary study was a part of the fifth Childhood and Adolescence Surveillance and Prevention of Adult Non-communicable disease (CASPIAN-V) in 2015. A total of 14,400 students 7-18 years and their parents living in 30 provinces in Iran were studied. A validated questionnaire of the World Health Organization on Global School-based Health Survey (WHO-GSHS) was used to measure the outcomes and socioeconomic variables. Family’s socioeconomic status (SES) was determined using principal component analysis (PCA). The crude and adjusted odds ratios (95% confidence interval (CI)) were estimated using multiple logistic regressions for each outcome.

**Results:**

A total of 14,274 students completed the study, of whom 50.6% were boys. Overall, the prevalence of bullying, being a victim, life dissatisfaction (LDS), and poor SRH among students was 35.6, 21.4, 21.1, and 19.0%, respectively. In multiple-logistic regression analysis (Adjusted OR, (95%CI), students with an illiterate father and mother (1.60, (1.25-2.04), 1.28, (1.03-1.61), unemployed father (1.58, (1.29-1.81)), and one-parent family (1.32, (1.05 – 1.64) had a higher odd of Poor-SRH. Besides, a family size larger than four members (1.14, (1.03-1.25), and low-SES (1.35, (1.15-1.56), and illiteracy of the mother (1.64, (1.30-2.08) had a direct association with LDS. Mother illiteracy also increased the odds of bullying (1.77, (1.45-2.16) and being a victim (1.58, (1.26-1.98).

**Conclusions:**

Some socioeconomic variables can be proposed as the statistically significant attribution of bullying and being a victim, LDS, and Poor-SRH in children and adolescents.

## Background

Among many topics that are important to any discussion of the interface between early life experience and total health, bullying in the school setting, life dissatisfaction (LDS), and self-rated health (SRH) are increasingly documented as predictors of instant and long-run health outcomes [1–3].

Although bullying was regarded as a regular part of children’s growing up [4], previous studies have explained a negative association between bullying and health outcomes [5, 6]. A study in the 21 rich European countries composing the Organization for Economic Co-operation and Development (OECD) verified that 1 out of 3 children had been bullied at least once during the last 2 months [7]. In Iran, a study on a sample of middle school students revealed that 79.6% of students are involved in bullying, and 81% are suffered bullying [8].

Life satisfaction (LS) is referred to the subjectively perceived quality of life according to the personal preferences of several life domains and the satisfaction in these domains [9]. LDS has been closely related to various negative personal, behavioural, psychological, and social outcomes [10, 11]. The majority of previous research on LS (or LDS) has been conducted primarily with adult participants [10], and relatively limited studies have investigated in childhood and adolescence [12].

SRH, as a single-item health predictor [1], is to ask about an individual’s perception of their overall health status [13]. Because of SRH consequences in adult life, exploring the SRH and its associated factors in early life may be of particular interest in health research. Previous studies suggest that conceptualizing health [14] and establishing healthy behaviors [15, 16] begin in early childhood and adolescence. Further, studies indicate that it can be regarded as the predictor of mortality [17], morbidity [18] and use of health care services [16, 19].

Given that, bullying, being bullied, LDS, and SRH have consistently been recognized as vital factors associated with positive growth, good health, and well-being in adulthood and understanding the socioeconomic variables attributed to them in childhood and adolescence is important. Limited information is available on the socio-economic determinants of childhood and adolescence self-rated health [20, 21], bullying, being a victim of violent behavior and LDS in school settings, especially in low and middle-income countries. Accordingly, we sought to investigate socio-economic determinants of bullying, being bullied, LDS, and SRH.

To the best of our knowledge, no study has been conducted in Iran to investigate the socioeconomic-related determinants of bullying, being bullied, LDS, and SRH, simultaneously, based on a national survey. Thus, using data from the Childhood and Adolescence Surveillance and Prevention of Adult Non- communicable disease” (CASPIAN) survey, the present study aimed to explore socioeconomic determinants including living area, family size, maternal and paternal education level and occupation status, family composition and family socioeconomic status on bullying, being bullied, LDS, and SRH among Iranian children and adolescents. As children and adolescents are often overlooked in health policy [21], this high generalizability study was conducted to allow policymakers to broaden their focus and to better develop early life-related health policies.

## Methodology

This multicenter cross-sectional study was the fifth survey of a surveillance program entitled “Childhood and Adolescence Surveillance and Prevention of Adult Non- communicable disease” (CASPIAN V) study (2015). The detailed methodology and executive procedures were described previously [22]; nonetheless, here, we point to essential subjects.

CASPIAN studies include national surveys on Iranian children and adolescents. The first CASPIAN survey took place in 2003 and was repeated every 2 or 3 years.

Using a multistage, stratified cluster sampling method, a total of 14,400 schoolchildren aged 7 to18 years were recruited from urban and rural areas across 30 provinces of Iran. Forty-eight clusters of schools were randomly selected in each province as the primary sampling unit. In each cluster, 10 students (and their parents) were randomly selected, resulting in 480 samples from each province. Using the proportional to size method and with an equal sex ratio, sampling was conducted according to the student’s place of residence (urban or rural) and level of education (primary and secondary).

### Questionnaires and measurements

Based on the World Health Organization- Global School-based student Health Survey (WHO-GSHS), two specific sets of questionnaires were developed for students and their parents. The student’s questionnaire was obtained from the WHO-GSHS that was translated into Persian. The validity and reliability of questionnaires have been confirmed previously. After explaining the study’s aims and executive procedure, written informed consent was obtained from all participants above the age of 16 and the parents/legal guardians of participants with 16 years of age and lower.

We developed a detailed protocol for the data collection procedures, including questionnaire filling techniques and physical examinations, and distributed it among team members. The data collection’s quality control and quality assurance were regularly monitored by the project’s Data and Safety Monitoring Board. Supervised by trained nurses, survey questionnaires were filled out anonymously. Physical examinations were performed by a trained team consisting of expert health care professionals.

### Definitions

#### Bullying

Bullying was assessed by asking: “During the past 3 months, how often did you bully someone at school?”. The possible choices were: “None” (considered as no), “One to two times” (considered as yes), “Two to three times” (considered as yes) and “Four times or more” (considered as yes) [23, 24].

#### Being bullied (victim)

According to the Global School-based Student Health Survey (GSHS) questionnaire on psychiatric distress and violent behaviors, victims were detected by asking, “During the past 3 months, how often did you get bullied at school?” The response choices were categorized as; “None” (considered as no), “One to two times” (considered as yes), “Two to three times” (considered as yes) and “Four times or more” (considered as yes) [23, 24].

#### Family socioeconomic status

The methods and variables for calculating the family SES were selected based on the categories approved in the Progress International Reading Literacy Study (PIRLS) for Iran [25]. The SES data was extracted from the parents’ questionnaire. The participants’ SES was determined based on the results of principle component analysis (PCA) variables of parents’ education, occupation, possessing a private car, their school type (public/private), home type (private/rented) and having a personal computer at home. The SES score was a weighted average of the SES variables that were summarized under one main component of “SES” score. A lower score corresponded to a lower SES. The calculated score was categorized into tertiles to define SES levels. The first tertile was considered as ‘low’, and the second and third ones as ‘middle’ and ‘high’ SES, respectively [24].

#### Life dissatisfaction (LDS)

To evaluate Life dissatisfaction (LDS), the participants were asked to express their degree of life satisfaction according to a tenth-point scale from 1 = very dissatisfied to 10 = very satisfied. Based on the results, a score below 6 was considered as Life dissatisfaction (LDS) [26, 27].

#### Self-rated health (SRH)

Students’ self-rated health (SRH) was assessed through questioning about “How would you describe your general state of health?” The response choices were categorized as; “perfect,” “good,” “moderate,” and “bad” [26, 27]. We summarized the responses as either ‘not poor’ (perfect or good) or ‘poor’ (moderate or bad) SRH.

### Statistical analysis

Continuous variables were expressed as mean and standard deviation (SD) and categorical variables as numbers (%). Chi-square test was used to compare the self-rated health, life satisfaction, and violent behaviors across the socioeconomic status variables. The association of socioeconomic status variables and violent behaviors, self-rated health, and life satisfaction were evaluated using different logistic regression models. Model I was a crude model (without adjustment); in model II, the association was adjusted for all socioeconomic status variables and age, simultaneously. All statistical analyses were conducted based on survey data analysis methods. Data were analyzed using the STATA package V.11.0 (Stata Statistical Software: Release 11. College Station, Texas, USA: StataCorp LP Package) and a *p*-value < 0.05 was considered significant.

## Results

A total of 14,274 students (50.6% boys, 49.4% girls) and at least one of their parents (out of 14,400, participation rate) completed the survey (participation rate: 99%). Table 1 shows students’ demographic and family characteristics, in total and by sex group.

**Table 1:** Socioeconomic characteristics and psychiatric distress according to sex: the CASPIAN-V study

Variable		Girl n (%)	Boy n (%)	Total n (%)	***p***-Value
Living area	Urban	5044 (71.6)	5150 (71.3)	10,194 (71.4)	0.657
	Rural	2002 (28.4)	2078 (28.7)	4080 (28.6)	
Family size	≤4	3291 (47.4)	3444 (48.3)	6735 (47.9)	0.310
	> 4	3645 (52.6)	3686 (51.7)	7331 (52.1)	
Maternal education level	College degree	776 (11.1)	747 (10.4)	1523 (10.8)	0.201
	Diploma and less	5012 (71.7)	5125 (71.5)	10,137 (71.6)	
	Illiterate	1202 (17.2)	1298 (18.1)	2500 (17.7)	
Paternal education level	College degree	997 (14.7)	895 (12.8)	1892 (13.7)	0.006*
	Diploma and less	4941 (72.7)	5211 (74.6)	10,152 (73.7)	
	Illiterate	858 (12.6)	876 (12.5)	1734 (12.6)	
Maternal occupation status	Employed	962 (13.7)	850 (11.8)	1812 (12.7)	0.001*
	Unemployed	6063 (86.3)	6352 (88.2)	12,415 (87.3)	
Paternal occupation status	Employed	6382 (91.3)	6550 (91.2)	12,932 (91.2)	0.833
	Unemployed	611 (8.7)	635 (8.8)	1246 (8.8)	
Family composition	Two parents	6586 (94.1)	6754 (94.1)	13,340 (94.1)	0.986
	Single parent	413 (5.9)	423 (5.9)	836 (5.9)	
Family SES	Low	2234 (33.3)	2325 (33.6)	4559 (33.5)	0.077
	Middle	2172 (32.4)	2343 (33.8)	4515 (33.1)	
	High	2297 (34.3)	2255 (32.6)	4552 (33.4)	

*SES* Socioeconomic status, *n* number

* a *P*-value under 0.05 was considered as statistically significant

Students’ mean ± SD age was 12.3 ± 3.2 years, with no significant difference between girls and boys. In girls, compared to boys, a higher percentage of mothers had a college degree (14.7% vs. 12.8%, *p* = 0.009) and were employed (13.7% vs. 11.8%, *p* < 0.001). There were no significant differences in other demographic and family characteristics between boys and girls. (Table 1).

Table 2 presents the frequency of bullying, being bullied, life dissatisfaction and poor health status according to sex and socioeconomic variables. Overall, 35.6% (95% CI: 34.9 – 36.4%) of students reported being a bully, and 21.4% (95% CI: 20.7-22.0%) of students were victims of bullying during the past 3 months. 21.1% (95% CI: 20.4 – 21.8) of our participants were dissatisfied with life and 19.0% (95% CI:18.4-19.7%) of them rated their health as poor.

**Table 2:** Frequency of bullying, being bullied, life dissatisfaction, and poor Self-rated health according to sex and socioeconomic variables: the CASPIAN-V study

Variable		Bullying n (%)	***p***-Value	Being bullied n (%)	***p***-Value	Life dissatisfaction n (%)	***p***-Value	Poor Self-rated health, n (%)	***p***-Value
Sex	Boy	2583 (35.9)	0.486	1573 (21.8)	0.137	1508 (21.0)	0.738	1345 (18.8)	0.538
	Girl	2477 (35.3)		1460 (20.8)		1488 (21.2)		1341 (19.2)	
Living area	Urban	3562 (35.1)	0.061	2128 (21.0)	0.106	2110 (20.8)	0.213	1929 (19.1)	0.589
	Rural	1498 (36.8)		905 (22.2)		886 (21.8)		757 (18.8)	
Family size	≤4	2382 (35.5)	0.915	1421 (21.1)	0.505	1270 (18.9)	< 0.001*	1279 (19.1)	0.955
	> 4	2579 (35.4)		1575 (21.6)		1693 (23.2)		1380 (19.1)	
Maternal education level	College degree	445 (29.4)	< 0.001*	304 (20.1)	< 0.001*	293 (19.4)	< 0.001*	246 (16.3)	< 0.001*
	Diploma and less	3591 (35.6)		2090 (20.7)		1978 (19.6)		1862 (18.6)	
	Illiterate	976 (39.2)		621 (24.9)		680 (27.3)		558 (22.6)	
Paternal education level	College degree	615 (32.7)	0.010*	347 (18.4)	0.001*	365 (19.4)	0.110	284 (15.1)	< 0.001*
	Diploma and less	3645 (36.1)		2243 (22.2)		2142 (21.2)		1963 (19.6)	
	Illiterate	591 (34.2)		357 (20.7)		382 (22.1)		354 (20.7)	
Maternal occupation status	Employed	603 (33.4)	0.036*	405 (22.5)	0.223	373 (20.7)	0.661	319 (17.8)	0.138
	Unemployed	4443 (36.0)		2620 (21.2)		2610 (21.1)		2360 (19.2)	
Paternal occupation status	Employed	4573 (35.5)	0.881	2771 (21.5)	0.151	2667 (20.7)	0.011*	199 (16.1)	0.005**
	Unemployed	444 (35.7)		246 (19.8)		296 (23.8)		2478 (19.4)	

*n* number

* a *P*-value below 0.05 was considered as statistically significant

All our outcomes including bullying, being bullied, life dissatisfaction, and poor Self-rated health were more frequent among individuals with low socioeconomic status (versus higher levels of SES), and those with an illiterate mother (versus other levels of education). (All of which with a *P*-values < 0.05). A higher percentage of individuals with a family size of four members and above (versus family size ≤4), single-parent family (versus two parents), and unemployed father (versus employed) were dissatisfied with their life. (All *p*-value < 0.05). Poor health status was less reported among those who their father had a college degree (versus below college degrees) or were employed (versus unemployed). (Both *P*-values < 0.05) Table 2.

A lower percentage of individuals with single parents (versus two parents) and a father with an academic education (versus below college degrees) described being bullied during the past 3 months. In addition, a lower percentage of students whose mother was employed (versus unemployed) or their father had a college degree (versus below college degrees), reported bullying someone during the past 3 months. (All *p*-value < 0.05) Table 2.

In the adjusted model of logistic regression analysis that all socioeconomic status variables and age were simultaneously in the model, (Table 3); higher odds of bullying someone were observed among students who lived in low SES (Adjusted OR, (95%CI): 1.21, (1.06-1.38)), two-parent family (1.39, (1.13-1.71)), and those whom their mothers had an education level ≤ diploma (1.46, (1.25-1.70)) or being an illiterate (1.77, (1.45-2.16)). Also, the odds of being the victim of violence (being bullied) were higher among students with a mother’s education level ≤ diploma (1.20, (1.01-1.43)) or being an illiterate (1.58, (1.26-1.98)), father education level below diploma (1.19, (1.03-1.38)), However, being bullied was 18% less among those who their mothers were unemployed (0.82, (0.71-0.95)) (Table 3).

**Table 3:** Associations of socioeconomic status variables with bullying and being bullied, the CASPIAN V study, logistic regression analysis

Variable		Bullying				Being bullied			
		Crude model		Adjusted model*		Crude model		Adjusted model**	
		OR (95% CI)	*P*-value	OR (95% CI)	*P*-value	OR (95% CI)	*P*-value	OR (95% CI)	*P*-value
Sex	Girl	Reference	–	Reference	–	Reference	–	Reference	–
	Boy	1.02 (0.95-1.09)	0.486	1.03 (0.95-1.10)	0.420	1.06 (0.98-1.15)	0.137	1.06 (0.97-1.15)	0.144
Living area	Urban	Reference	–	Reference	–	Reference	–	Reference	–
	Rural	1.07 (0.99-1.15)	0.061	1.07 (0.99-1.16)	0.073	1.07 (0.98-1.17)	0.106	1.03 (0.94-1.13)	0.489
Family size	≤4	Reference	–	Reference	–	Reference	–	Reference	–
	> 4	0.99 (0.93-1.06)	0.915	0.95 (0.84-1.08)	0.06	1.02 (0.94-1.11)	0.505	0.95 (0.86-1.04)	0.278
Maternal education level	College Degree	Reference	–	Reference	–	Reference	–	Reference	–
	Diploma And Less	1.32 (1.18-1.49)	< 0.001*	1.46 (1.25-1.70)	< 0.001*	1.04 (0.91-1.19)	0.557	1.20 (1.01-1.43)	0.035*
	Illiterate	1.55 (1.35-1.77)	< 0.001*	1.77 (1.45-2.16)	< 0.001*	1.32 (1.13-1.54)	< 0.001*	1.58 (1.26-1.98)	< 0.001*
Paternal education level	College Degree	Reference	–	Reference	–	Reference	–	Reference	–
	Diploma And Less	1.16 (1.04-1.29)	0.005	1.01 (0.92-1.1)	0.80	1.26 (1.11-1.43)	< 0.001*	1.19 (1.03-1.38)	0.02*
	Illiterate	1.07 (0.93-1.23)	0.315	1.05 (0.94-1.24)	0.38	1.15 (0.97-1.36)	0.089	0.81 (0.65-1.02)	0.09
Maternal occupation status	Employed	Reference	–	Reference	–	Reference	–	Reference	–
	Unemployed	1.11 (1.00-1.24)	0.036*	0.89 (0.78-1.01)	0.082	0.92 (0.82-1.04)	0.223	0.82 (0.71-0.95)	0.010*
Paternal occupation status	Employed	Reference	–	Reference	–	Reference	–	Reference	–
	Unemployed	1.00 (0.89-1.14)	0.881	0.92 (0.79-1.08)	0.344	0.89 (0.77-1.04)	0.151	0.9 (0.76-1.09)	0.16
Family composition	Two Parents	1.13 (0.97-1.31)	0.107	1.39 (1.13-1.71)	0.002*	1.28 (1.07-1.54)	0.007*	0.94 (0.75-1.19)	0.649
	Single Parent	Reference	–	Reference	–	Reference	–	Reference	–
Family SES	Low	1.22 (1.12-1.33)	< 0.001*	1.21 (1.06-1.38)	0.004*	1.12 (1.01-1.24)	0.021*	1.14 (0.98-1.33)	0.076
	Middle	1.10 (1.01-1.20)	0.021*	1.10 (1.00-1.22)	0.050	0.99 (0.89-1.10)	0.894	1.02 (0.91-1.15)	0.644
	High	Reference	–	Reference	–	Reference	–	Reference	–

*OR* Odds ratio, *CI* Confidence interval, *SES* Socioeconomic status.

* a *P*-value below 0.05 was considered as statistically significant

**In the adjusted model, all socioeconomic status variables and age are simultaneously in the model

As presented in Table 4, students with an illiterate mother (1.64, (1.30-2.08)), low SES family (1.35, (1.15-1.56)), and family size > 4 (1.14, (1.03-1.25)), had higher odds of LDS. However, living in a two-parent family indicated an indirect association with life dissatisfaction (0.76, (0.61-0.95)). Regardless of maternal and paternal education levels less than college (≤ diploma & illiterate), and the father’s unemployment, all observations were significantly associated with higher odds of poor health status. (*P*-value < 0.05) (Table 4).

**Table 4:** Associations of socioeconomic status variables with life dissatisfaction and Poor Self-rated health: the CASPIAN V study, logistic regression analysis

Variable		Life dissatisfaction				Poor Self-rated health			
		Crude model		Adjusted model**		Crude model		Adjusted model**	
		OR (95% CI)	***P***-value	OR (95% CI)	***P***-value	OR (95% CI)	***P***-value	OR (95% CI)	***P***-value
Sex	Girl	Reference	–	Reference	–	Reference	–	Reference	–
	Boy	0.99 (0.92 - 1.07)	0.738	1.00 (0.92-1.08)	0.940	0.97 (0.89-1.05)	0.538	0.97 (0.89-1.06)	0.535
Living area	Urban	Reference	–	Reference	–	Reference	–	Reference	–
	Rural	1.06 (0.97-1.16)	0.213	1.04 (0.94-1.15)	0.480	0.97 (0.88-1.07)	0.589	0.98 (0.89-1.08)	0.762
Family size	≤4	Reference	–	Reference	–	Reference	–	Reference	–
	> 4	1.30 (1.21 - 1.41)	< 0.001*	1.14 (1.03-1.25)	0.011*	0.99 (0.91-1.08)	0.955	0.98 (0.89-1.09)	0.826
Maternal education level	College Degree	Reference	–	Reference	–	Reference	–	Reference	–
	Diploma And Less	1.02 (0.89 - 1.18)	0.825	1.12 (0.93-1.33)	0.237	1.17 (1.01-1.35)	0.033*	1.26 (1.04-1.51)	0.015*
	Illiterate	1.59 (1.35 -1.85)	< 0.001*	1.64 (1.30-2.08)	< 0.001*	1.49 (1.27-1.76)	< 0.001*	1.60 (1.25-2.04)	< 0.001*
Paternal education level	College Degree	Reference	–	Reference	–	Reference	–	Reference	–
	Diploma And Less	1.12 (0.99 - 1.27)	0.082	1.07 (0.94-1.22)	0.28	1.36 (1.19-1.56)	< 0.001*	1.28 (1.09-1.50)	0.002*
	Illiterate	1.19 (1.01 - 1.39)	0.043*	1.05 (0.88-1.25)	0.66	1.46 (1.23-1.73)	< 0.001*	1.28 (1.03-1.61)	0.03*
Maternal occupation status	Employed	Reference	–	Reference	–	Reference	–	Reference	–
	Unemployed	1.03 (0.92 - 1.16)	0.661	0.87 (0.75-1.01)	0.060	1.10 (0.96-1.25)	0.138	0.91 (0.78-1.07)	0.275
Paternal occupation status	Employed	Reference	–	Reference	–	Reference	–	Reference	–
	Unemployed	1.20 (1.04 - 1.37)	0.011*	1.05 (0.88-1.25)	0.578	1.26 (1.07-1.47)	0.005*	1.58 (1.29-1.81)	< 0.001*
Family composition	Two -Parent	0.81 (0.69-0.94)	0.007*	0.76 (0.61-0.95)	0.014*	0.95 (0.79-1.13)	0.579	0.87 (0.68-1.11)	0.276
	Single - Parent	Reference	–	Reference	–	Reference	–	Reference	–
Family SES	Low	1.47 (1.39-1.61)	< 0.001*	1.35 (1.15-1.56)	< 0.001*	1.14 (1.03-1.27)	0.012*	0.95 (0.81-1.12)	0.567
	Middle	1.03 (0.92-1.15)	0.622	1.06 (0.94-1.21)	0.331	1.04 (0.94-1.16)	0.403	0.95 (0.84-1.08)	0.483
	High	Reference	–	Reference	–	Reference	–	Reference	–

*OR* Odds ratio, *CI* Confidence interval, *SES* Socioeconomic status.

* a *P*-value below 0.05 was considered as statistically significant

**In the adjusted model, all socioeconomic status variables and age are simultaneously in the model

There were no significant associations between other socioeconomic variables with the assessed outcomes and the crude and adjusted odds ratios were generally similar (as shown in Tables 3 and 4).

## Discussion

In this study, by using a nationally representative dataset from CASPIAN V, we focused on SES determinants of bullying, being bullied, LDS and poor SRH among students aged 7 to 18 years in Iran. The findings imply that among socioeconomic variables, the mother’s illiteracy increased the odds of bullying, being bullied, LDS and poor SRH among students. Further, the father’s illiteracy and low level of education, and father’s unemployment increase the odds of poor SRH. Moreover, family size > 4, single parenthood and low-SES were associated with life dissatisfaction. To our knowledge, this is the first attempt to declare socioeconomic attributions related to bullying, being bullied, LDS and poor SRH in the early life simultaneously in Iran. It is important since the influencing variables in early life pave the way for health status and well-being later in life [28].

To our knowledge, there is still no evidence on all socioeconomic factors associated with bullying in Iran. However, our finding contributes to the existing literature suggesting the role of family characteristics including parental education as an important factor related to the risk of bullying [29–31]. We find that the mothers’ illiteracy and low maternal education are risk factors for students to bully and get bullied. Our result is in keeping with previous findings that show low parental education level has been associated with an increased risk of bullying [30, 32, 33]. Jansen et al., using longitudinal data from a subsample of the Tracking Adolescents’ Individual Lives Survey (TRAILS) in the Netherlands found that the children of parents with low educational levels (as a marker of low socioeconomic status in families) were more likely to bully, get bullied, or bully and get bullied simultaneously [30]. In Germany, Von Marées and Petermann, using a cross-informant approach showed that low parental education levels significantly increased the chances of being a bully/victim among primary school children [33]. Nordhagen et al., in a cross-sectional comparative study conducted in the five Nordic countries, showed that children of parents with low education seemed to be bullied more often than counterparts with high education [32]. Nevertheless, some other studies revealed no statistically significant association between parental education level and bullying among children and adolescents [6, 31]. About the negative effects of low parental education on bullying one can assume that parent’s low educational level as so-called risk marker [33], can raise risk factors such as authoritarian parenting style, family stress, parental conflicts, poor communication with parents, lack of involvement and warmth in family [34] and household material deprivation [31] which are related to bullying. It was also implying that parents with low education are less involved with school activities and policies that has been a risk factor for bullying or being a bullied [35].

Our findings regarding socioeconomic variables and LDS are consistent with those of other studies [2, 10, 36–38]. Adolescent LDS is related to various early life experiences in the family environment [39]. Out of these early experiences, family composition including family size (i.e. number of adults living in the home) is significantly related to LDS. However, by reviewing the literature, no studies were found in other countries that explored the association of family size with LDS. Nonetheless, in Iran, with the information of 13,486 students aged 6-18 years, Kelishadi et al., found that LDS is significantly higher in students with > 4 family members [37]. It is suggested that this finding in crowded families may be related to the continuing struggle for achieving household financial resources and emotional support, low rate of room per capita, limited share of food and more conflicts between siblings leading to a low level of life satisfaction (LS) among family members. We found that families with single parents increased the likelihood of dissatisfying life among students. This finding is consistent with previous studies that showed living with single parents had an inverse relationship with LS [37, 40, 41]. In the United States, Zulling et al., with the use of the statewide data from the Youth Risk Behavior Survey (YRBS) indicated that both white male and female adolescents who reported living with two parents were significantly less likely to report LDS [41]. In our study, The mother’s illiteracy was a risk factor for LDS. A study among European American, African American, Chinese American, Mexican American, and Dominican American adolescents showed that LS was positively correlated with parental education [42]. Moreover, Crede et al. in a sample of German high school students, reported that although fathers’ education did not moderate the relationship between students’ LS scores and academic achievement, mothers’ education did [43]. Nevertheless, another study in the USA reported no statistical significance between the mother’s and father’s level of education and LS [44]. Our finding regarding family SES and LDS was consistent with different studies that show a significant direct association between low SES and LDS [36, 45, 46]. One study included a sample of 2823 Croatian high school students, authors concluded that adolescents’ perception of their family’s economic status had a modest positive correlation to LS [46]. Chappal et al. showed that students in the low SES group reported lower LS compared to middle/high SES students [45]. In Iran, Mirmoghtadaee et al. showed that compared to high family SES, low family SES increases the odds of low LS [26]. Kelishadi et al. also reported the same findings [37]. However, some studies examining the role of SES with respect to LS reported no difference in disfavor of lower SES students [47, 48].

With regard to the SLR of students with illiterate mothers and fathers, having a father with a low level of education and unemployment had a greater association with poor SRH, which was consistent with some other studies [1, 49, 50]. Goodman et al., recruiting 1179 adolescents from Princeton City School District, demonstrated that lower parent education was associated with fair–poor SRH [49]. Results from 22 European and North American countries showed that the most deprived students (i.e. students with a low level of parental education and occupation) had an odds ratio nearly three times higher than the least deprived students for self-rated poor health [50].

### Strengths and limitations of the study

The study’s main strengths lie in the quantity and quality of the data, collected in a large nationally representative sample size and designed and conducted according to the standardized questionnaire of the World Health Organization on Global School-based Health Survey (WHO-GSHS). As data were drawn from a cross-sectional study, causal interpretations should be made with caution. In fact, attribution of causality might be better discovered with prospective longitudinal research in the future studies. Moreover, the sample size was very large in our study. We acknowledge that this may lead to significant findings that may be of dubious relevance.

## Conclusions

According to the findings, some socioeconomic variables can be proposed as the statistically significant attributions of bullying, being bullied, LDS and poor-SRH in children and adolescents. Namely, parental education, father’s occupation, family size, and family’s SES can be taken into account in anti-bullying initiatives and programs related to LS and SRH promotion.

## Data Availability

The data that support the findings of this study is belonged to health surveillance system of ministry of health (MOH), therefore the availability of data are restricted However data upon request from the corresponding authors and with permission of MOH is available.
